# Cognitive biases can affect moral intuitions about cognitive enhancement

**DOI:** 10.3389/fnsys.2014.00195

**Published:** 2014-10-15

**Authors:** Lucius Caviola, Adriano Mannino, Julian Savulescu, Nadira Faulmüller

**Affiliations:** ^1^Department of Experimental Psychology, University of OxfordOxford, UK; ^2^Department of Philosophy, University of BernBern, Switzerland; ^3^Oxford Uehiro Centre for Practical Ethics, University of OxfordOxford, UK; ^4^Oxford Centre for Neuroethics, University of OxfordOxford, UK; ^5^Department Values, Technology and Innovation, Delft University of TechnologyDelft, Netherlands

**Keywords:** cognitive enhancement, rationality, cognitive bias, attitudes, de-biasing, moral intuitions, brain function augmentation

## Abstract

Research into cognitive biases that impair human judgment has mostly been applied to the area of economic decision-making. Ethical decision-making has been comparatively neglected. Since ethical decisions often involve very high individual as well as collective stakes, analyzing how cognitive biases affect them can be expected to yield important results. In this theoretical article, we consider the ethical debate about cognitive enhancement (CE) and suggest a number of cognitive biases that are likely to affect moral intuitions and judgments about CE: status quo bias, loss aversion, risk aversion, omission bias, scope insensitivity, nature bias, and optimistic bias. We find that there are more well-documented biases that are likely to cause irrational aversion to CE than biases in the opposite direction. This suggests that common attitudes about CE are predominantly negatively biased. Within this new perspective, we hope that subsequent research will be able to elaborate this hypothesis and develop effective de-biasing techniques that can help increase the rationality of the public CE debate and thus improve our ethical decision-making.

## Cognitive enhancement and biased reasoning

The enhancement of cognitive brain functions by means of technology has become a reality. Recent research has demonstrated that various pharmaceuticals can have—at least modest—performance-enhancing effects in healthy individuals (for reviews, see Repantis et al., 2010; Husain and Mehta, 2011). In line with these findings, the off-label use of pharmacological substances like methylphenidate (e.g., Ritalin-^®^) or modafinil (e.g., Provigil-^®^) seems to be prevalent, in particular among students (Herman-Stahl et al., 2007; Smith and Farah, 2011; Dietz et al., 2013), and professionals in very responsible jobs like physicians (Franke et al., 2013). It has even been argued that the use of such substances might become an implicit requirement for individuals in certain professions (e.g., Maslen et al., in press). Non-pharmacological methods of cognitive enhancement (CE), such as gene therapy or neural implants, could become more widely used in the future (Bostrom and Sandberg, 2009). Even though there is considerable use of CE, recent research shows that the general public has strongly negative attitudes regarding the introduction and use of CE (for a review, see Schelle et al., 2014).

In this article, we argue that these concerns are partly driven by pervasive cognitive biases. A bias is a systematic deviation from a standard of rationality (Baron, 2005), an error frequently committed by the human mind. We use the definition of “rationality” common in cognitive science, referring to normative models of accurate belief formation (Foley, 1987; Audi, 2001) and optimal decision-making in order to achieve one’s goal (Dawes, 1998). (Ir)rationality is therefore relative to the goal of having accurate beliefs or to goals in general. Thus, while some cognitive patterns may be suboptimal with regard to a particular goal, they may be perfectly optimal with regard to another. For illustration, consider “status quo bias”, which we will address in the following section. If, upon reflection, an agent consciously and stably places some intrinsic value on the status quo, then in no sense can the agent be said to irrationally prefer the status quo (i.e., to make a cognitive mistake in doing so). However, the available evidence suggests that human agents do not, upon reflection, intrinsically care about the status quo, or at least to a significantly lesser degree than their immediate intuitions would have it: the experiments documenting our intuitive status quo preference (e.g., Samuelson and Zeckhauser, 1988) are interesting because they reveal a fact about our intuitive decision-making which our reflection disapproves of and is thus irrational. Also, nearly all action-guiding moral theories have an account of when the status quo should be modified, which is why the general bias towards it will tend to bring about suboptimal moral outcomes (c.f. Bostrom and Ord, 2006). Furthermore, if by “moral” decision-making we mean decision-making that is optimal from an “altruist” or “impartial” perspective, then non-related goals such as status quo preservation will lead to irrationality, too. Hence, it is justifiable to classify the status quo bias and the other biases we are discussing below as, indeed, biases.

The aim of this paper is to offer a new perspective on the debate about CE by identifying cognitive biases that affect our common intuitions and moral reactions. We will first give a rough overview of the academic debate and the public’s view of CE and then discuss a number of potential biases that are likely to influence widespread judgments about CE.

In the academic debate about CE, opinions of critics (often bioconservatives) and supporters (often transhumanists) are quite sharply divided. Critics often question its medical safety, considering the potential side effects and unknown long-term health risks (Healey and Rayner, 2008). Critics also see CE as a threat to human autonomy (Habermas, 2003), authenticity (Elliott, 2004) humility and solidarity (Sandel, 2004) and equality and fairness (Fukuyama, 2004). Proponents, on the other hand, focus on the potential positive aspects and potentials of CE. For instance, Savulescu (2006) points out that the introduction of CE could help decrease unfair natural differences in abilities. Bostrom (2008) emphasizes the enormous positive leverage effect that CE could generate in the future. He speculates that a 1% increase in all scientists’ cognitive performance “would amount to an indirect contribution equal to 100,000 times what the average scientist contributes” (p. 2). Others note that CE is not relevantly different from traditional forms of enhancement, such as information technology (Harris, 2010).

Recent research reviewing the general public’s attitudes towards CE has shown that lay people share many of the critics’ concerns about CE (Schelle et al., 2014). Participants from samples of students or parents, for example, fear that safety is not ensured (Forlini and Racine, 2012; Partridge et al., 2013). They think that CE could lead to peer pressure and thus undermine our autonomous decisions (Forlini and Racine, 2009). Also, they worry that unequal distribution of CE would result in an unfair advantage of a privileged few (Fitz et al., 2013). These views are often reflected in exaggerated reports by the media and might even lead to severe aversive social consequences for users (Faulmüller et al., 2013). The Guardian, for example, has described CE substances as “capitalism’s wonder-pills”, which “turn you into the closest human approximation there is to a machine” (Mahdawi, 2012).

We believe that the common concerns about CE are justified at least to some extent, and a greater scientific understanding of CE is required in order to draw confident conclusions (Maslen et al., 2014). Most importantly, long-term safety of CE is not currently ensured (c.f. Husain and Mehta, 2011) and is being called into question (e.g., Urban and Gao, 2014). Nevertheless, we speculate that the public’s views on CE are negatively biased. While the involved biases do not necessarily undermine any specific argument for or against CE—these would need to be evaluated on their philosophical and scientific merits—they might partially explain the general opposition to CE as well as the selective focus on certain arguments and exaggeration of their relative weight. It is not unusual for human cognition to selectively search for certain classes of arguments and systematically neglect or undervalue certain others (c.f. Kunda, 1990 on “motivated reasoning”)—a bias that might affect the judgments of both opponents and proponents of CE. Biases may lead people to come up with a set of arguments that expresses sound considerations but is significantly incomplete or weighted wrongly. We thus propose that in order to fully explain prevalent opposition to CE, it is necessary to look into the role biases play in the CE context. Awareness of these biases is a precondition of rational public debate about CE, which in turn is needed for developing and establishing optimal social and legal regulations.

Empirical research into biases over the last four decades has shown that human reasoning is very prone to systematic irrational patterns, i.e., cognitive biases (e.g., Tversky and Kahneman, 1974), especially when the subject matter is as complex, novel, abstract and ideologically loaded as is the use and regulation of CE (Cosmides and Tooby, 1992; Kahan et al., 2013). We argue that a number of cognitive biases well-documented in psychology and behavioral economics partly explain prevalent negative attitudes towards CE. While we believe that attitudes towards CE are predominantly negatively biased, there may also be biases leading us to irrationally favor CE. We will indicate some biases likely to affect judgments about CE and illustrate why and how they may be impairing our judgments in this context. Thus, our aim is to suggest several hypotheses about potential irrational sources of the prevalent (negative or positive) attitudes towards CE. Some of the biases on our below list have been studied in behavioral economics, such as status quo bias, loss aversion, risk aversion, scope insensitivity. The others—omission bias, nature bias, and optimistic bias—have been addressed more in psychology and philosophy. Our application of the knowledge about biases to the CE debate is comparatively novel, as is the application to ethical issues in general. We hope to offer a valuable perspective as a contribution to more rational public debate on controversial ethical issues involving new practices or technologies. Since ethical questions often involve high stakes, this endeavor can be expected to yield important results for public ethical debate.

## Potential cognitive biases

### Status quo bias

Status quo bias describes the tendency to prefer the current state of affairs over a change even if a change would result in better expected outcomes. For instance, Samuelson and Zeckhauser (1988) presented participants with hypothetical choice tasks about financial investment, which either were defined with a clear status quo or not. Participants were significantly more likely to choose the option designated as the status quo compared to the same option that was not labeled the status quo. Numerous further experiments reliably demonstrate this effect (e.g., Kahneman et al., 1991). It seems plausible that popular aversion to CE is partly due to status quo bias: it may partly result from the pure fact that CE constitutes a novelty. Historically, new ideas and technologies have often encountered strong aversion and opposition at first (Jay, 1981; Weil and Rosen, 1995), but became accepted at a later point in time. Coffee—a traditional form of CE—provides an instructive example: it was first considered an unacceptable drug and even forbidden in some countries (Weinberg and Bealer, 2001; Cowan, 2005). Bostrom and Ord (2006) have already drawn attention to the role status quo bias likely plays in the debate about CE. They also suggest a de-biasing tool—the *Reversal Test*—designed to expose and overcome status quo bias. If we believe that the enhancement of certain cognitive abilities will have negative consequences, we should consider the reverse: if an increase in intelligence is judged as negative, would a decrease in intelligence be judged favorable? If not, we are committed to claiming that our current level of intelligence is at an at least local optimum. This is, of course, possible but would require further justification. Obviously, it is an empirical question whether the introduction of CE actually results in net positive consequences or not. In case it would, however, status quo bias still exerts a psychological force against its endorsement. Empirical research can potentially expose the prevalence of status quo bias by, for example, observing whether people come to a different conclusion after reflecting on the rationale of the Reversal Test and applying it to the context of CE.

### Loss aversion

Loss aversion describes the tendency to weigh losses more than gains (Kahneman and Tversky, 1984). In monetary contexts, for example, loss aversion results in a stronger dissatisfaction after losing $1 than satisfaction after gaining $1 (Kahneman and Tversky, 1984). Whether something is perceived as a loss or as a gain depends on how the decisional situation is framed: one can either “get a $1 discount” or “avoid a $1 surcharge”. Loss aversion can act on its own, but may also be a source of status quo bias (Bostrom and Ord, 2006). Maybe we recognize positive as well as negative consequences that the introduction of CE likely entails, such as the potential benefits due to enhanced intelligence on the one hand and the fear of increased unfairness in society due to unequal access to CE on the other hand (Fitz et al., 2013). But due to loss aversion, we likely tend to exaggerate the weight of the negative consequences relative to the positive ones.

### Risk aversion

Risk aversion describes the tendency to undervalue an option that is less certain compared to a more certain option with equal expected outcome value (Kahneman and Tversky, 1984). Since preserving the status quo usually involves less uncertainty than a change does, risk aversion may be a further source of status quo bias, and it can act on its own as well. Consider a gamble where you have the option A of winning $1000 with a chance of 85% or the option B of winning $800 for sure. Most people prefer option B to A. However, option A has a higher expected value of 0.85 ^*^ $1000 = $850 and is therefore the rational decision if one values money linearly. As we are constantly faced with uncertainty, we are in fact dealing with such gambles all the time. We may be reluctant to introduce CE simply because its expected consequences involve probabilities that deviate more strongly from 100% and 0% than do the ones of the status quo. For example, methylphenidate almost certainly improves memory (for a meta-analysis, see Repantis et al., 2010), but there is a chance of long-term adverse effects (King et al., 2006). Though the expected utility calculation may favor the use of methylphenidate in certain cases, people are likely to retain a preference against it due to risk aversion and a resulting “precautionary principle” heuristic.

### Omission bias

Omission bias describes the tendency to judge decisions differently depending on whether the same outcome is brought about through an act or an omission (Spranca et al., 1991). More specifically, people consider harms that have been caused by action worse than equal harms caused by omission. Ritov and Baron (1990) observed that parents often show reluctance to vaccinate their children. The expected harm of non-prevented disease is much greater than the expected harm of vaccination (Gangarosa et al., 1998), but parents seem to overvalue the expected harm caused by the vaccination because it is the result of their action. Similarly, we may fear that the introduction of CE is likely to or may cause harm. However, CE may also prevent harms from occurring and thus refraining from introducing may be a harmful omission, i.e., a missed opportunity for reducing harm. Due to our general omission bias, we are likely to systematically overvalue the harm of introducing CE compared to the harm resulting from not introducing it. For example, people might believe that the use of CE substances by medical doctors potentially causes active harm by inducing sleep disorders (Partridge et al., 2013)—but they might neglect or underestimate the potential positive consequences of doctors’ increased ability to focus, i.e., the potential negative consequences of inaction. Rejecting CE may lead to greater harms suffered by more people overall. This question cannot be settled by unreflective intuition but requires open—un-biased—scientific investigation.

### Scope insensitivity

Scope insensitivity occurs when people don’t assign appropriate weight to the quantity of a decisional option (Kahneman, 2000; Desvousges et al., 2010). For example, a study has demonstrated that people are willing to pay the same amount of money to either help 9,000 people or 90,000 people who are at risk (Kahneman and Knetsch, 1992; Baron and Greene, 1996). This is irrational if one’s goal is to help people and have everyone count the same, for the money one is willing to pay should then scale linearly with the number of people affected. It seems that our ability to intuitively represent such large numbers and their relations correctly is quite limited. In the context of CE, a probable implication is that we are not giving appropriate weight to the enormously high number of individual decisions, people (and generations) potentially affected by the consequences of introducing CE and thus to the importance and priority of the CE issue.

### Nature bias

Several studies on peoples’ attitudes towards CE have revealed that natural CE substances are seen as less harmful and less ethically problematic than artificial ones (e.g., Bergström and Lynöe, 2008). For example, in a study with university students, participants were more likely to consider the use of artificial CE substances morally wrong than herbal ones (Scheske and Schnall, 2012). These judgments seem to be impaired by a form of nature bias (c.f. the fallacy of *appealing to nature*), i.e., a tendency to view the “natural” as good and the “unnatural” as bad. Given that many examples of “natural and bad” (e.g., diseases) as well as “unnatural and good” (e.g., medicine) things exist, it is highly questionable whether tracking the “natural” tracks what people (would) actually assign value to, and it is clear that it is not intrinsically morally relevant according to the “altruistically/impartially optimal decision-making” criterion.

### Optimistic bias

Some biases may also be pushing people towards an overly optimistic evaluation of the consequences of introducing CE. An example is the optimistic bias described by Chapin and Coleman (2009), which causes people to underestimate the possibility of negative outcomes. Examples include people underestimating the risk of becoming a victim of crime, of losing money in the markets, or of getting lung cancer after smoking (Weinstein and Klein, 1996). In the context of CE, people might underestimate the dangers, such as negative side effects, that CE could entail.

An additional source of optimistically skewed views of CE might result from biased reduction of cognitive dissonance by people who are already using forms of CE. When confronted with falsificatory information about CE, such as documented health concerns, users might experience discomfort—cognitive dissonance—and be likely to selectively adopt beliefs that justify their behavior (Festinger, 1957).

Thus, there are potential biases leading to an overly positive attitude towards CE. Overall, however, it seems there are more well-documented highly prevalent biases likely to cause irrational aversion to CE than biases in the opposite direction.

## Conclusion

We have aimed to offer and motivate the perspective that biases are likely to impair judgments about CE, mostly in the negative direction. We are aware that the above list of biases is incomplete and speculative and hope that empirical research will further elaborate on our suggestions. It must be emphasized that “biases” often do provide sensible decisional rules of thumb—after all, many of them seem to have proven to be successful heuristics for our evolutionary ancestors. However, the practical scope of this argument is very limited first because our current environment is different from our evolutionary environment, and second because the metaphorical goals of our genes that have been served by some “bias-heuristics” need not coincide with our goals (Stanovich, 2004). Nevertheless, the existence of such biases cannot alone undermine the soundness of any objection to CE. But they can explain why people, including policy makers, may take an unreflective or one-sided position on such debates. More generally, many of our moral intuitions about controversial practices are likely to be influenced by such cognitive biases. Not only do we require psychological interventions such as remedial heuristics, we require good ethical argument and relevant scientific evidence to identify which of our moral intuitions are justified, and which are not.

We believe the CE debate would benefit from research investigating the role cognitive biases play in judgments about CE and from the subsequent development of techniques that help people judge the relevant issues in a less biased way. These techniques should be simple heuristics (Larrick, 2004) that are easily applicable in the context of CE. Bostrom and Ord (2006) *Reversal Test* or Savulescu (2007) *Loss/Gain Heuristic* are examples of such heuristics. Psychological research could test whether people’s attitudes towards CE change after applying de-biasing techniques, i.e., by being made aware of the biases potentially impairing their judgments. If they do change, we have reason to assume that cognitive biases play a role in the current attitudes towards CE.

## Conflict of interest statement

The authors declare that the research was conducted in the absence of any commercial or financial relationships that could be construed as a potential conflict of interest.
